# Biomechanical analysis of the tandem spinal external fixation in a multiple-level noncontiguous lumbar fractures model: a finite element analysis

**DOI:** 10.3389/fbioe.2024.1395197

**Published:** 2024-06-19

**Authors:** Huarong Chen, Yu Kang, Yiguo Yan, Hu Wang, Wen Peng, Yijia Liao, Mingxiang Zou, Zhun Xu, Xizheng Song, Wenjun Wang, Cheng Wang

**Affiliations:** ^1^ The First Affiliated Hospital, Department of Spine Surgery, Hengyang Medical School, University of South China, Hengyang, Hunan, China; ^2^ Central People’s Hospital of Zhanjiang, Zhanjiang, Guangdong, China; ^3^ The Third Affiliated Hospital of Sun Yat-sen University, Department of Spine Surgery, Sun Yat-sen University, Guangzhou, Guangdong, China; ^4^ Luoyang Orthopedic-Traumatological Hospital of Henan Province (Henan Provincial Orthopedic Hospital), Luoyang, Henan, China

**Keywords:** tandem spinal external fixation, finite element analysis, multilevel noncontiguous spinal fracture, biomechanics, thoracolumbar fracture

## Abstract

**Objective:**

This study aimed to investigate the biomechanical characteristics of the tandem spinal external fixation (TSEF) for treating multilevel noncontiguous spinal fracture (MNSF) using finite element analysis and provide a theoretical basis for clinical application.

**Methods:**

We constructed two models of L2 and L4 vertebral fractures that were fixed with the TSEF and the long-segment spinal inner fixation (LSIF). The range of motion (ROM), maximum stresses at L2 and L4 vertebrae, the screws and rods, and the intervertebral discs of the two models were recorded under load control. Subsequently, the required torque, the maximum stress at L2 and L4 vertebrae, the screws and rods, and the intervertebral discs were analyzed under displacement control.

**Results:**

Under load control, the TSEF model reserved more ROM than the LSIF model. The maximum stresses of screws in the TSEF model were increased, while the maximum stresses of rods were reduced compared to the LSIF model. Moreover, the maximum stresses of L2 and L4 vertebrae and discs in the TSEF model were increased compared to the LSIF model. Under displacement control, the TSEF model required fewer moments (N·mm) than the LSIF model. Compared to the LSIF model, the maximum stresses of screws and rods in the TSEF model have decreased; the maximum stresses at L2 and L4 in the TSEF model were increased. In the flexion condition, the maximum stresses of discs in the TSEF model were less than the LSIF model, while the maximum stresses of discs in the TSEF model were higher in the extension condition.

**Conclusion:**

Compared to LSIF, the TSEF has a better stress distribution with higher overall mobility. Theoretically, it reduces the stress concentration of the connecting rods and the stress shielding of the fractured vertebral bodies.

## Introduction

The multilevel noncontiguous spinal fracture (MNSF) is a unique type of spinal fracture characterized by two or more fractured vertebral bodies separated by at least one normal vertebral body (Korres et al., 1981; Powell et al., 1989; Iencean, 2002; Lian et al., 2007). MNSF is primarily caused by high-energy injuries, such as traffic accidents and high falls (Wittenberg et al., 2002; Bensch et al., 2006; Kanna et al., 2016). Extreme pain, spinal instability, deformity, and neurological dysfunction are the frequent clinical features of MNSF. The most common treatment for MNSF is the LSIF system using open posterior surgery (Takami et al., 2017). However, it has been reported that the long-segment screw-rod fixation (usually five segments or more) exhibits many inherent defects, including loss of range of motion (ROM), stress concentration on the screws or rods, and stress shielding of the fractured vertebral bodies.

**FIGURE 1:**
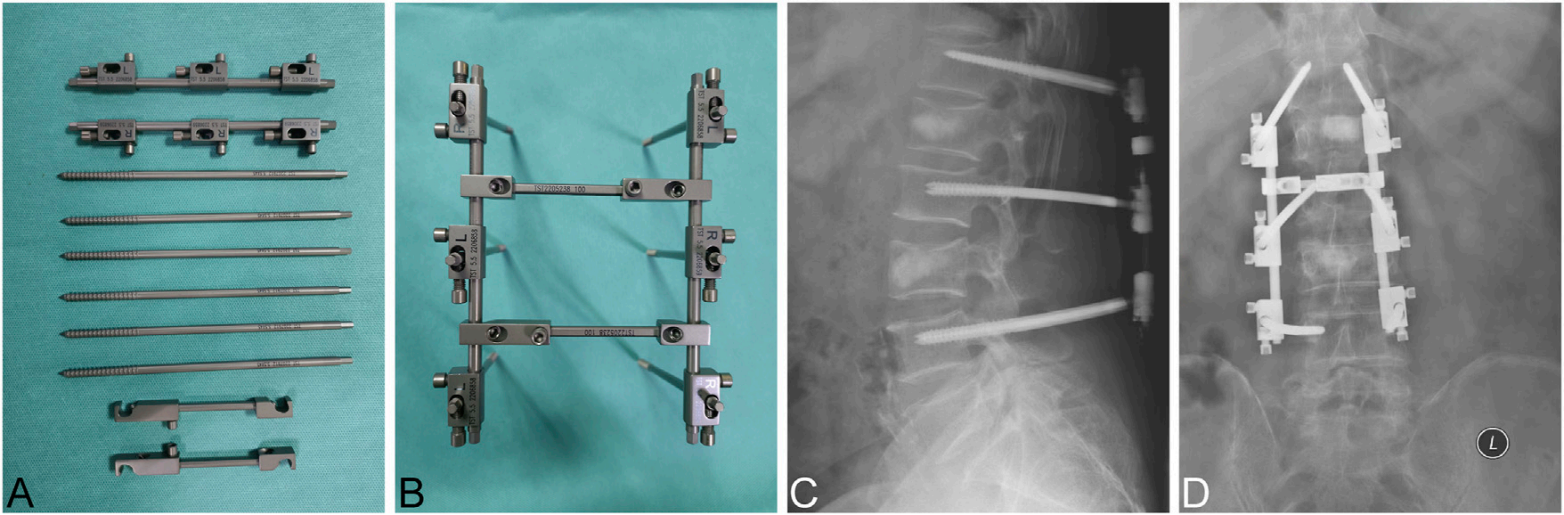
Clinical application of TSEF **(A)** Components of TSEF. **(B)** The overall diagram of TSEF. **(C,D)** X-Ray images of clinical application of TSEF. The bone substitute showed in this figure was Bicera™ (Wiltrom Ltd, Taiwan.).

The external spinal fixation systems we utilized in previous studies have achieved satisfactory clinical outcomes in treating single-segment thoracolumbar fractures (Wang et al., 2011; Song et al., 2014; Wang C. et al., 2018). Spinal external fixation is a minimally invasive surgical technique that reduces the fractured vertebral body percutaneously. Generally, the external fixation is removed 3 months after the operation, thus reducing the risk of complications caused by prolonged rigid fixation (Wang et al., 2011). Inspired by the single-segment external fixation, we developed the tandem spinal external fixation (TSEF) for MNSF, which demonstrated no neurological symptoms (Figures 1A–D). The TSEF has many clinical advantages compared to the traditional long-segment spinal inner fixation (LSIF), but the potential mechanic characteristics of TSEF are yet to be explored.

Finite element analysis (FEA) is a method for analyzing complex solid and structural mechanics systems and has been widely used in orthopedic biomechanics research. Complex spinal fractures can be reconstructed using FEA based on computed tomography (CT) technology. Moreover, the biomechanical characteristics of spinal implants can be well revealed using FEA (Xu et al., 2014a; Liao et al., 2017; Liao et al., 2022). This study evaluated the biomechanical characteristics of the TSEF in treating MNSF using FEA and provided the corresponding theoretical basis for the clinical application in MNSF.

## Material and methods

### Construction and validation of finite element model of L1–L5 lumbar spine

**FIGURE 2:**
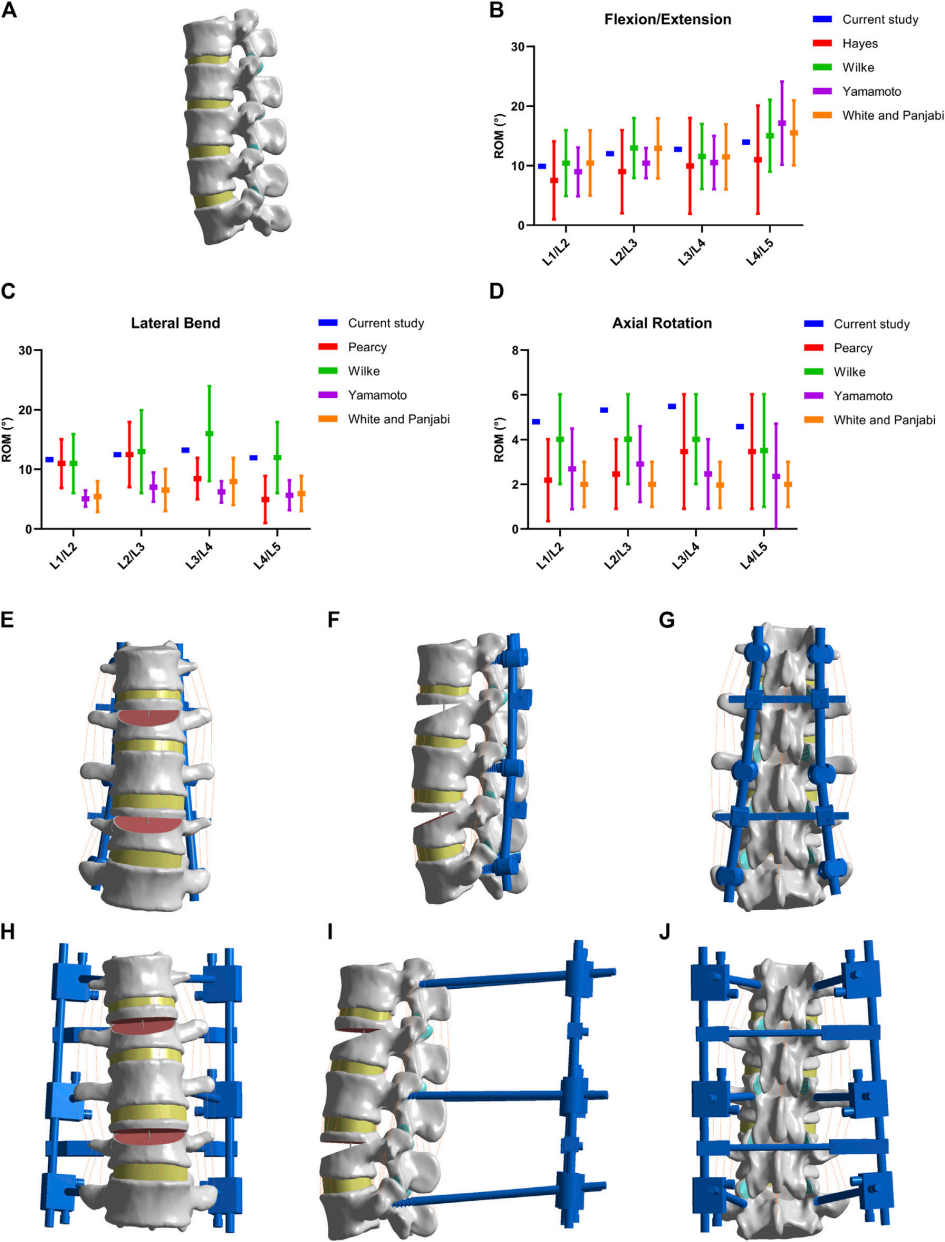
Models establishment and validation. **(A)** Normal lumbar model. **(B–D)** Validation of normal lumbar validation. **(E–G)** Model of MNSF fixed by LISF. **(H–J)** Model of MNSF fixed by TSEF.

The DICOM format files for the finite element model construction (L1–L5 lumbar spine) were obtained from a healthy volunteer (26 years old male weighing 71 kg and height of 173 cm) using a 64-slice spiral CT. After thresholding segmentation, dynamic growth, mask editing, and Boolean manipulation, the three-dimensional (3D) contour model of L1–L5 was extracted using the software Mimics 20.0 (Materialize, Belgium). Slices of the contour model were collected using Geomagic 12 (Geomagic Inc., United States) and then processed by smoothing, grinding, denoising, surface construction, and solidification. SolidWorks 2015 (Dassault, France) was used to reconstruct the intervertebral disc (matrix and nucleus pulposus) and articular surface. The reconstructed model vertebral bodies consisted of cortical bone, cancellous bone, and endplates. The intervertebral discs consisted of the nucleus pulposus and fibrous ring (3:7). The articular cartilage was set at 0.3 mm thickness, and the upper and lower articular cartilages were in frictional contact with a friction coefficient of 0.1. Seven paravertebral ligaments, including the anterior longitudinal ligament, posterior longitudinal ligament, ligamentum flavum, interspinous ligament, supraspinous ligament, capsular ligament, and intertransverse process ligament, were modeled. Intact L1–L5 lumbar model material and characteristics were assigned based on previous studies (Wang H. et al., 2018; Han et al., 2022), as shown in Table 1. The mesh sizes of 3 mm, 2 mm, 1.5 mm, 1 mm, and 0.5 mm were meshed using Ansys workbench 18.0 (Ansys, United States). The 5% change indicated that the mesh was converged. According to the mesh convergence results shown in Table 2, a final mesh size of 1 mm was selected for the subsequent analysis of this study (Figure 2A). The L1–L5 lumbar spine finite element model (FEM) was validated by comparing it with previous study data (White and Panjabi, 1976; Pearcy et al., 1985; Hayes et al., 1989; Yamamoto et al., 1989; Wilke et al., 1997) (Figures 2B–D).

**TABLE 1:** Material properties of the finite element model (Wang H. et al., 2018; Han et al., 2022).

Component name	Young’s modulus(MPa)	Poisson’s ratio	Cross-sectional area(mm^2^)
Cortical bone	12,000	0.3	-
Cancellous bone	100	0.3	-
Injured canellous bone	10	0.3	-
Cartilage	10	0.4	-
Bony endplate	1,000	0.4	-
Nucleus pulposus	1	0.499	-
Annulus fibrosus	4.2	0.3	-
ALL	20	0.3	63.7
PLL	20	0.3	20
LF	19.5	0.3	40
ISL	11.6	0.3	40
SSL	15	0.3	30
TL	58.7	0.3	3.6
CL	32.9	0.3	60
Instruments	110,000	0.3	-

ALL, anterior longitudinal ligament; PLL, posterior longitudinal ligament; LF, ligamentum flavum; ISL, interspinous ligament; SSL, supraspinal ligament; TL, transverse ligament; CL, capsular ligament.

**TABLE 2:** Parameters for mesh convergence.

Size of mesh (mm)	Element	Node	Stress on vertebra (MPa)
0.5	2,229,305	3,157,044	20.09
1	693,194	1,030,241	20.08
1.5	311,639	493,005	19.19
2	279,010	302,980	18.15
3	90,017	173,045	16.86

### Model construction of MNSF

The L2 and L4 vertebral bodies were selected to simulate the fracture. The finite element model of MNSF was constructed as described in the previous literature (Liu et al., 2018; Zhou et al., 2020). Briefly, a line parallel to the upper endplate was made at 15% of the anterior edge of the L2 vertebral body to the posterior edge of the vertebral body. Then a diagonal line was made from the intersection of the line and the posterior edge of the vertebral body to 55% of the anterior edge of the vertebral body as a triangle. Then, the triangular part of the vertebral body was removed. The same procedure was performed on the L4 vertebral body.

### Model construction of MNSF fixed with LSIF and TSEF

Models of LSIF and TSEF were constructed using SolidWorks 2015 (Dassault, France). The diameter specifications of the pedicle screws and connection rods of LSIF were 6.5 mm and 6 mm, respectively. The diameter specifications of the pedicle screws and connection rods of TSEF were 5.5 mm and 5 mm, respectively. Pedicle screws were transpedicular and placed in L1, L3, and L5 vertebral bodies. Screws should be parallel to the upper endplate and implanted beyond the anterior 1/3 of the vertebral body. The pedicle screw holders of LSIF were partially attached to the bone surface, and the connecting rods were fixed with the screw holder U-groove. The predial apparatus fixed the connecting rods of TSEF on the body surface of L1–L5. The final normal model included 693,194 units and 1,030,241 nodes, the LSIF model included 694,386 units and 1,058,953 nodes, and the TSEF model included 739,105 units and 1,121,711 nodes (Figures 2E–J). Given that the bone and implant remained within the range of small deformations under body load, a linear elastic constitutive model was assumed for both the implant and the bone components in the presenting analysis (Xu et al., 2014b). The linear elastic constitutive equation was described as followed:

σ=E·ε

(σ indicates stress, E indicates elasticity modulus, ε indicates deformation)

### Control mode and boundary condition

Load control [500 N preload and 7500 N mm motion torque (Müller et al., 2021; Han et al., 2022)] was applied to the upper surface of the L1 vertebral body, while the bottom of the L5 vertebral body was fully fixed and restrained. The range of motion (ROM) of different models was recorded under six working conditions: flexion and extension, left and right lateral flexions, and left and right rotations. The maximum stresses on screws, rods, fractured vertebral bodies, and intervertebral discs in internal and external fixation models were compared and analyzed.

The maximum ROMs in flexion and extension conditions of the TSEF model were defined as the boundary conditions. The preload of 500 N was applied as described previously (Liao et al., 2022). An increasing motion torque was applied to the models gradually, and the values of motion torque that the two different models required to achieve boundary conditions were recorded and analyzed. Meanwhile, the maximum stresses of the screws, rods, fractured vertebral bodies and intervertebral discs were recorded.

## Results

### Load control

#### Range of motion (ROM)

**FIGURE 3:**
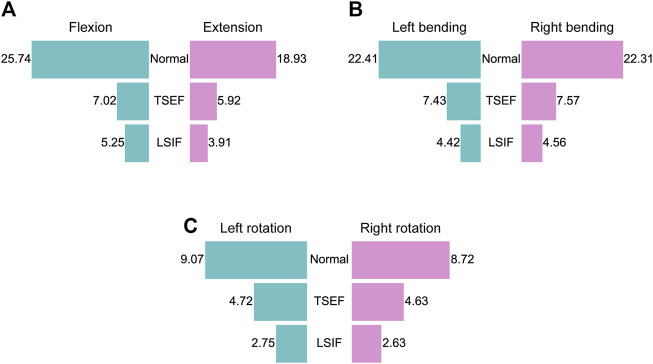
Restriction of two fixations on lumbar mobility under the six conditions. **(A)** ROM of the normal, TSEF and LSIF models under flexion and extension conditions. **(B)** ROM of the normal, TSEF and LSIF models under left and right bending conditions. **(C)** ROM of the normal, TSEF and LSIF models under left and right rotation conditions.

The ROM of flexion, extension, left bending, right bending, left rotation and right rotation were 25.74°, 18.93°, 22.41°, 22.31°, 9.07°, and 8.72° for the normal model; were 7.02°, 5.92°, 7.43°, 7.57°, 4.72°, and 4.63° for the TSEF model; were 5.25°, 3.91°, 4.42°, 4.56°, 2.75°, and 2.63° for the LSIF model (Figures 3A–C). The ROM of the TSEF model was smaller than that of the normal lumbar model, which was 27.3%–53.1% of the normal lumbar model. The ROM of the LSIF model was smaller than that of the normal lumbar model, which was 19.7%–30.3% of the normal lumbar model. Thus, both TSEF and LSIF models significantly restricted the ROM of the lumbar spine.

#### Von mises stress of pedicle screws and connecting rods

**FIGURE 4:**
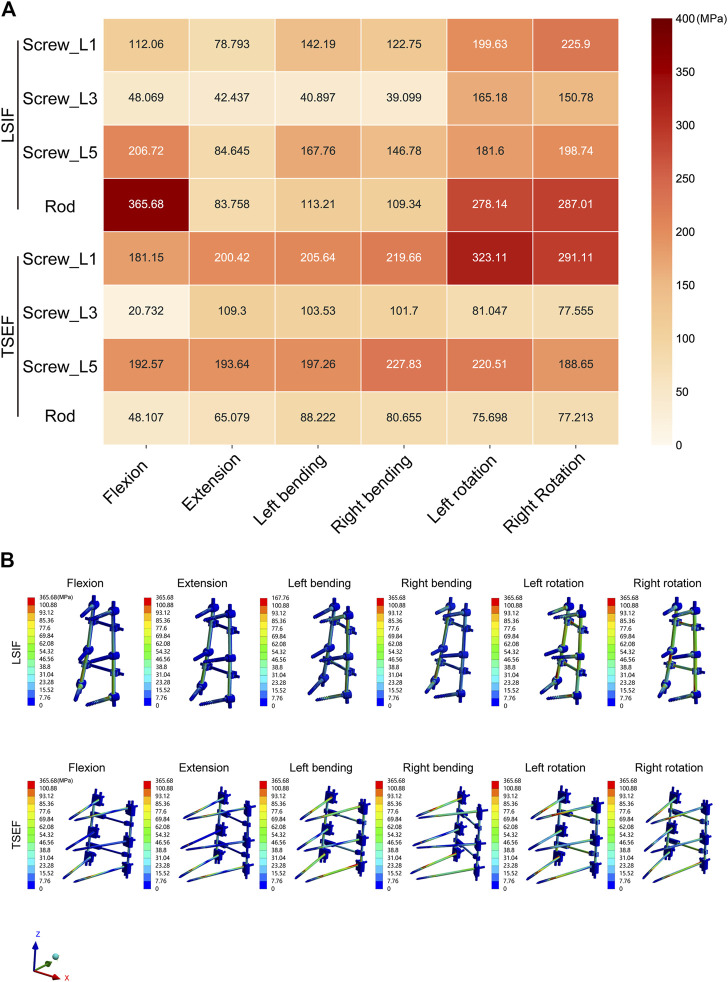
Maximum von mises stress analysis of LSIF and TSEF under the six conditions. **(A)** Heatmap of the Maximum von mises stress on LSIF and TSEF under the six conditions. **(B)** Cloud plots of the Maximum von mises stress on LSIF and TSEF under the six conditions.

The maximum values of stress and cloud plots of pedicle screws and connecting rods of TSEF and LSIF models under the six operating conditions are shown in Figure 4. The maximum von Mises stress value of pedicle screws of LSIF and TSEF models were observed in the left and right rotation conditions. The maximum von Mises stress value of the LSIF model in the right rotation was 225.9 MPa, and the maximum von Mises stress value of the TSEF model in the left rotation was 323.11 MPa. The maximum stress of pedicle screws of the TSEF model was greater than that of the LSIF model except for flexion. The maximum stress of the connecting rod of the LSIF model was 365.68 MPa during the flexion, and that of the connecting rod of the TSEF model was 88.22 MPa during the left flexion. The maximum stress on connecting rod of the TSEF model was less than that of the LSIF model under all conditions. In the TSEF model, the uppermost and lowermost screws bore the higher stress. In the LSIF model, the uppermost and lowermost screws and the connecting rods primarily shared the stress.

#### Maximum von mises stress of the fractured vertebral body

**FIGURE 5:**
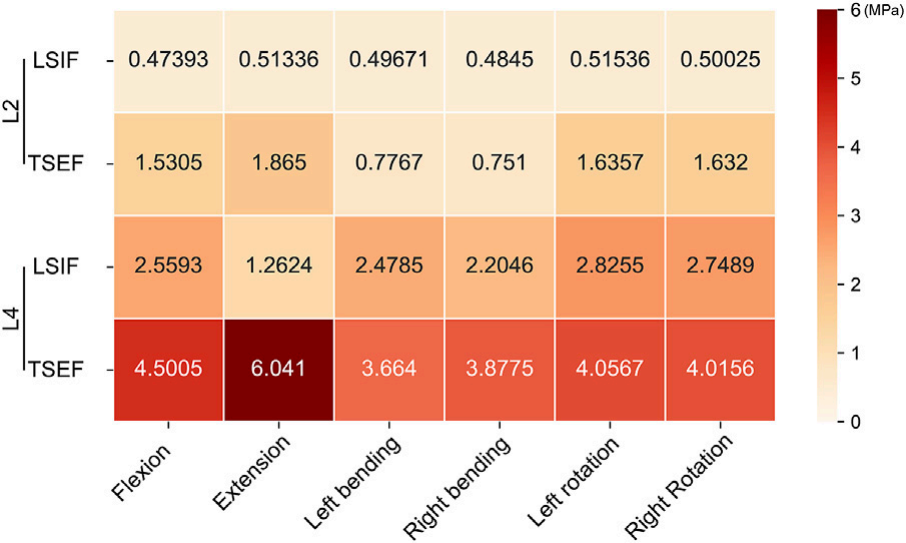
Maximum von mises stress on L2 and L4 in LSIF and TSEF under the six conditions.

Under all working conditions, the maximum von Mises stress values of the L4 fractured vertebral body of the two groups of models were higher than that of the L2 fractured vertebral body. Moreover, the maximum von Mises stress values of the L2 and L4 fractured vertebral bodies of the TSEF model were higher than that of the LSIF model (Figure 5).

#### Maximum von mises stress of intervertebral disc

**FIGURE 6:**
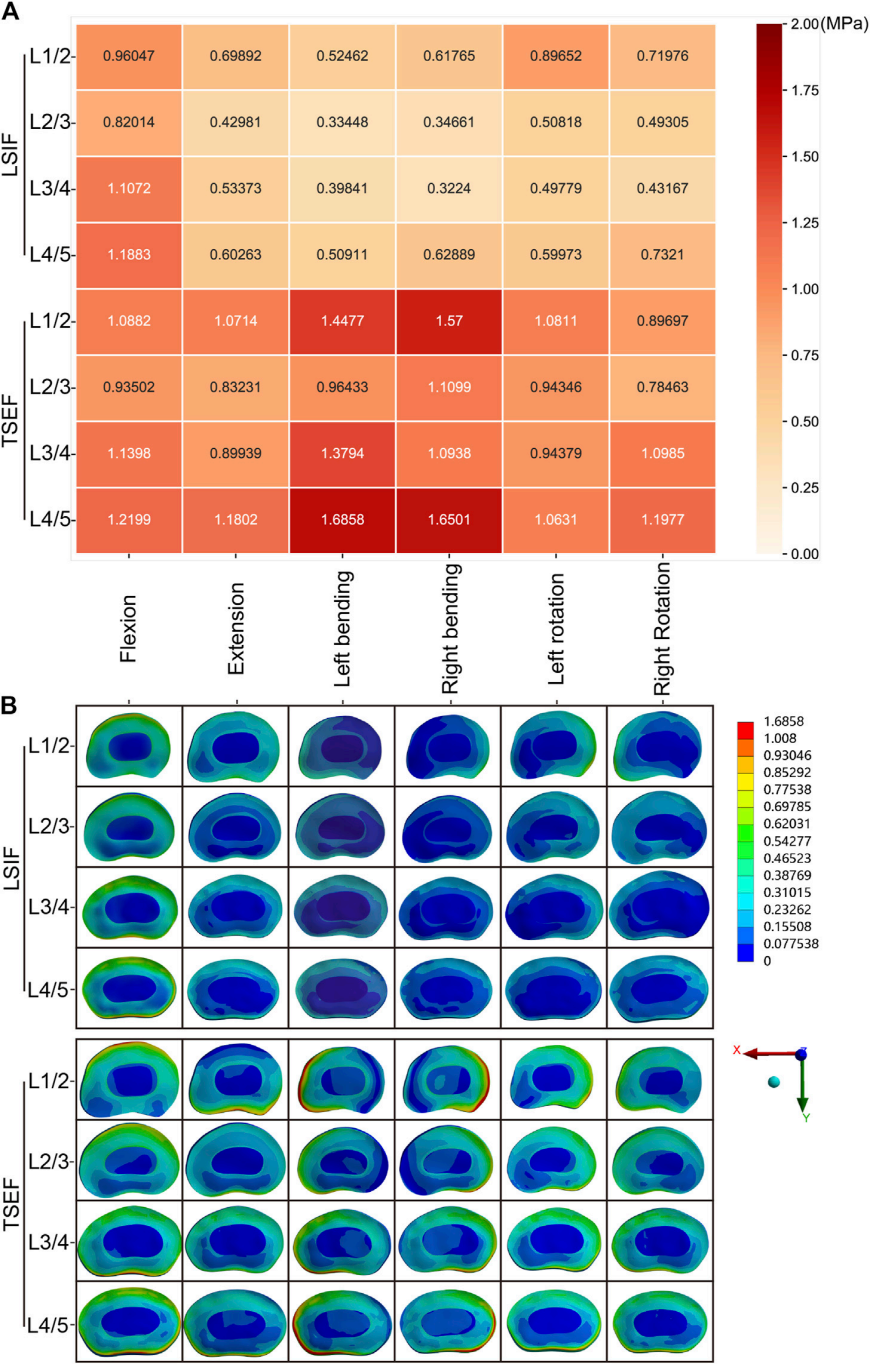
Maximum von mises stress analysis of intervertebral discs under the six conditions. **(A)** Heatmap of the Maximum von mises stress on intervertebral discs under the six conditions. **(B)** Cloud plots of the Maximum von mises stress on intervertebral discs under the six conditions.

In all working conditions, the maximum von Mises stress values of the intervertebral discs in the TSEF model were higher than that of the corresponding discs in the LSIF model (Figure 6A). In flexion, the maximum stress of the disc in the TSEF model was 1.22 MPa (L4/L5), while the LSIF model was 1.19 MPa (L4/L5). In extension, the maximum stress of the disc in the TSEF model was 1.18 MPa (L4/L5), while the LSIF model was 0.70 MPa (L1/L2). In left bending, the maximum stress of the disc in the TSEF model was 1.69 MPa (L4/L5), while the LSIF model was 0.52 MPa (L1/L2). In right bending, the maximum stress of the disc in the TSEF model was 1.65 MPa (L4/5), while the LSIF model was 0.63 MPa (L1/L2). In left rotation, the maximum stress of the disc in the TSEF model was 1.08 MPa (L1/2), while the LSIF model was 0.90 MPa (L1/L2). In the right rotation, the maximum stress of the disc in the TSEF model was 1.20 MPa (L4/L5), while the LSIF model was 0.73 MPa (L4/L5). The cloud plots of the intervertebral discs of TSEF and LSIF models under the six operating conditions are shown in Figure 6B.

### Displacement control

#### Motion torque required for the same ROM

**FIGURE 7:**
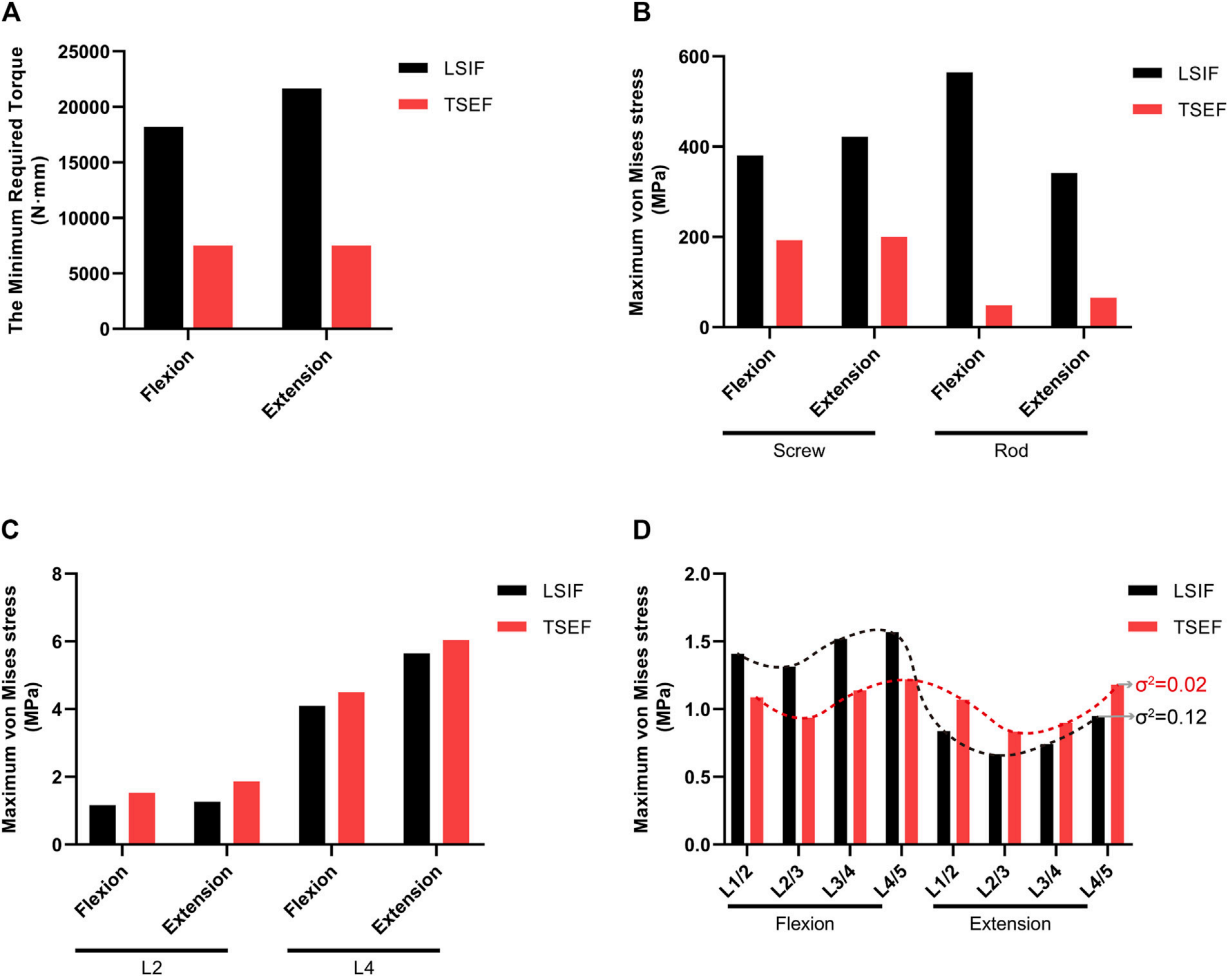
Comparison of LSIF and TSEF under displacement control. **(A)** Required torque for the two fixations to reach the ROM of flexion 7.02° and extension 5.29°. **(B)** Maximum von mises stress on screws and rods of the LSIF and TSEF models under the displacement control. **(C)** Maximum von mises stress on fractured vertebra bodies (L2 and L4) in LSIF and TSEF under the displacement control. **(D)** Maximum von mises stress on discs in LSIF and TSEF under the displacement control. σ^2^: variance.

Under load control, the maximum ROM of the TSEF model was 7.02° in flexion and 5.92° in extension, which was the target value of displacement control. When the flexion activity reached 7.02°, the required torque for TSEF and LSIF were 7500 N·mm and 18,200 N·mm, respectively. When the extension activity reached 5.29°, the TSEF and LSIF models required 7500 N · mm and 21650 N · mm, respectively. The required motion torque of the LSIF model was higher than that of the TSEF model (Figure 7A).

#### Maximum von mises stress of pedicle screws and connecting rods

Under displacement control, the maximum stress of screws and connecting rods in the LSIF model was higher than that in the TSEF model in flexion (screw: 380.51 vs. 192.57 MPa; rod: 564.73 vs. 48.11 MPa). The maximum stress of screws and connecting rods in the LSIF model was higher than that in the TSEF model in extension (screw: 421.81 vs. 200.42 MPa; rod: 341.84 vs. 65.08 MPa) (Figure 7B).

#### Maximum von mises stress of fractured vertebral bodies

Under displacement control, the maximum stress of fractured vertebral bodies in the TSEF model was higher than that in the LSIF model in flexion (L2: 1.53 vs. 1.16 MPa; L4: 4.50 vs. 4.10 MPa). The maximum stress of fractured vertebral bodies in the TSEF model was higher than that in the LSIF model in the extension condition (L2: 1.87 vs. 1.27 MPa; L4: 6.04 vs. 5.65 MPa) (Figure 7C).

#### Maximum von mises stress of intervertebral discs

Under displacement control, the maximum stress of intervertebral discs in the TSEF model was less than that in the LSIF model in flexion condition (L1/L2: 1.09 vs. 1.41 MPa; L2/L3: 0.94 vs. 1.31 MPa; L3/L4: 1.14 vs. 1.52 MPa; L4/L5: 1.22 vs. 1.57 MPa); the maximum stress of intervertebral discs in the TSEF model was higher than that in the LSIF model in extension condition (L1/L2: 1.07 vs. 0.84 MPa; L2/L3: 0.83 vs. 0.67 MPa; L3/L4: 0.90 vs. 0.74 MPa; L4/L5: 1.18 vs. 0.95 MPa). The variance was calculated to represent the degree of dispersion of the two sets of data. Variance in the TSEF model was less than that in the LSIF model (Figure 7D).

## Discussion

There are different ways to set a follower load on the spine model. The follower load parameters can be modified based on the target segments. In the study of Elmasry et al., the thoracolumbar spine models (T12-L2) were loaded with a 400 N follower load and 5000 N·mm moments (Elmasry et al., 2018). When selecting the lumbar spine as the subject of research, it is common practice to apply a follower load of 500 N at the L1 level, with a torque set at 7500 N·mm (Müller et al., 2021; Han et al., 2022). Therefore, the presenting lumbar model was loaded with a 500 N follower load and 7500 N·mm moments.

Under load control, the maximum ROM of the internal fixation model in six conditions ranged from 19.7% to 30.3% of the normal lumbar spine model. The result indicated that the entire lumbar spine was effectively fixed with the traditional long-segment spinal inner fixation, but the mobility of the lumbar spine was significantly limited. The maximum ROM of the TSEF was 27.3%–53.1% of the normal lumbar spine model. The TSEF model preserved more ROM compared to the LSIF model without losing stability. The overall ROM in the TSEF model was increased by 38.6%–75.2% compared to the LSIF model. Therefore, this might reduce the incidence of complications resulting from mobility loss of the thoracolumbar spine to a certain extent.

Additionally, we found that the maximum stress of the pedicle screw in the external fixation model was higher than that in the internal fixation model under all conditions except the flexion. However, the values of the stresses in the external fixation model were far below the fatigue threshold of 550 MPa, indicating the low risk of breakage of TSEF. Moreover, based on our previous experience, it is not adequate to analyze the stress of the screw-rob system purely using load control because the activity of the human spine is primarily achieved through displacement control (Liao et al., 2022). In order to simulate and analyze the specific stress situation of the nail bar system, we adopted the maximum activity of the external fixed model under flexion and extension conditions as the target. We achieved the same activity by increasing the load of the internal fixation model. In this case, we found that the load applied by the internal fixation system reached 242.7% and 288.7% of the initial load. Therefore, the patients treated with the LSIF system need to exert more force using the low back muscles to complete the same amplitude of movement under the same motion situation. Theoretically, it increases the burden on the tendon ligament complex of the lower back following the surgery.

At the same time, under the displacement control, the maximum stress of the LSIF model was significantly higher than that before, and the stress of the screw and the connecting rod was significantly higher than the maximum stress of the TSEF model. Even under the flexion condition, the stress value of the rod reached 564.73 MPa, which indicated there might be a fracture risk (Zhou et al., 2020). Our results reversely proved that the safety of the TSEF was significantly better than that of the long-segment screw-rod internal fixation system, and the safety of the screw-rod system was not significantly reduced due to the increase in its ROM. According to the clinical use of external spinal fixation, we usually remove the external fixation system 3 months after surgery, so no screw breakage occurs in clinical practice.

This study analyzed and compared the maximum stress of the fractured vertebral bodies. The maximum stress of the L4 vertebral body of the two models was higher than that of the L2 vertebral body under both load control and displacement control. Under load control, the maximum stress of the L2 and L4 fractured vertebral bodies of TSEF increased by 222.94% and 75.849% relative to LSIF in flexion condition, and increased by 263.29% and 378.53% relative to LSIF in extension condition. However, when utilizing displacement control, which more accurately replicates real motion, the increments were significantly decreased. Under displacement control, the maximum stress of the L2 and L4 fractured vertebral bodies of TSEF increased by 31.53% and 9.82% relative to LSIF in flexion condition, and increased by 47.11% and 6.98% relative to LSIF in extension condition. Hence, it is our contention that the modest rise in stress under displacement control conditions should not, in theory, have a significant impact on the healing or exacerbation of the fracture. Previous studies have shown that rigid fixation may lead to stress shielding (Frost, 2004), which is not conducive to fracture healing, and long-segment fixation further increases this risk (Seçer et al., 2015). A certain degree of micromotion and stress stimulation may promote fracture healing (Uhthoff et al., 2006; Bottlang et al., 2010; Augat et al., 2021; Duan and Lu, 2021; Travascio et al., 2021). According to experimental data, the maximum stress in the fractured vertebrae of the external fixation model increased slightly compared to the internal fixation model, and the TSEF model preserved more ROM compared to the LSIF model. Theoretically, the external fixation system has a potential in reducing the stress shading of the fractured vertebrae and promoting early healing of the fractured vertebrae. Similarly, the vast majority of the fractured vertebrae had already reached clinical healing at 3 months using external fixation in our clinical setting. Under load control, the maximum stress of the intervertebral disc of the TSEF was smaller than that of the LSIF in flexion condition, while greater than that of the LSIF in extension condition. The variance, reflecting the discrete degree of a data set, was smaller in TSEF compared to LSIF. It indicated that TSEF exhibited a better biomechanical characteristic regarding discs stress distribution compared to LSIF.

In this study, we demonstrated the function of two fixation systems in the body through load control and displacement control, but there were some limitations. The lumbar model was constructed using the radiographic data from a healthy male volunteer, which discounted the influence of the morphological heterogeneity in lumbar spine. The cortical and cancellous bone were defined as homogeneous materials with different Young’s modulus, and the intervertebral discs and cartilage are modeled as elastic elements. However, the morphology and density of cancellous bone play a significant role in determining the biomechanical properties of the vertebral body, with variations in bone density due to gender and age influencing surgical decision-making (Al-Barghouthi et al., 2020; Garay et al., 2022). Additionally, complex muscle interactions and the viscoelastic behavior of disc fibers and ligaments are easily ignored in the dics computational model (Volz et al., 2022). Hence, the current model exhibits certain constraints in accurately replicating real motion scenarios. The incorporation of multiple models, encompassing a wide range of parameters including cancellous bone morphology, age, gender, and intervertebral disc metabolism characteristics, will significantly enhance its applicability.

On the other hand, the uniformed screws were utilized in the construction of two models (6.5 mm for LSIF, 5.5 mm for TSEF). If each screw model were constructed based on the SD/PW ratio (screw diameter/pedicle width) as previously documented (Solitro et al., 2019; Solitro et al., 2024), the model would more accurately reflect the variability in dimensions of the lumbar segments. In a study regarding the screw pullout load reported by Giovanni F. Solitro et al., given that the friction coefficient and shear stress are critical for screw pullout analysis, a layer surrounding the screw was modeled to include a failure for shear ata value of 1, and the bone-screw interface was modeled as surface-to-surface contact with a coefficient of friction of 2 (Solitro et al., 2024). In the prsenting study, we focused on ROM of the lumbar spine and stresses on model elements in the TSEF and LSIF models, hence the bone-screw interface was defined as binding contact without modeling surrounding screws (Xu et al., 2014b).

The advancement of orthopedic implants has led to the exploration and application of emerging metal materials and screw insertion techniques. Michal Szczodry and others introduced a novel technique known as Increased Cortical Purchase (ICP) that demonstrates comparable accuracy to conventional methods and has the potential to enhance the long-term stability of pedicle screw fixations (Szczodry et al., 2018). A study conducted by Patrick A. Massey and co-authors presented biomechanical evidence suggesting that nitinol memory rods utilized in a posterior construct exhibit similar compression properties to titanium rods, but possess higher torsional failure load and torsional toughness (Massey et al., 2021). Sweetu Patel and others conducted research to develop and assess a biomedical Ti6Al4V rod aimed at enhancing the stability of the bone-rod interface (Patel et al., 2015). In order to broaden the scope of applicability of this model, which is now confined to the direct implantation mode of conventional titanium alloy implants, it is suggested that factors such as material properties, surface coating, and screw placement method be incorporated into the model construction parameters.

## Conclusion

MNSF is a unique type of spinal trauma commonly caused by high-energy injuries, and its global incidence was 1.6%–16.7% (Seçer et al., 2015). The surgical treatment of MNSF with spinal instability is still controversial (Lian et al., 2007; Seçer et al., 2015; Cho and Kim, 2019). Some studies reported treating MNSF using a long-segment screw-rod inner fixation system that fixed range could cover the entire lumbar spine (L1–L5) (Lian et al., 2007; Seçer et al., 2015; Cho and Kim, 2019). However, the traditional long-segment spinal inner fixation (LSIF) would lead to extensive soft‐tissue damage, loss of spinal ROM, stress concentration on fixation, stress shielding of fractured vertebral bodies, and adjacent segment degeneration (ASD) (Bastian et al., 2001; Park et al., 2004; Uhthoff et al., 2006; Bottlang et al., 2010; Augat et al., 2021; Duan and Lu, 2021). We developed the TSEF based on our previous research and the single-segment external spinal fixation system to deal with the above problems. The TSEF has many advantages in managing MNSF due to its simpler structure, smaller surgical incision, faster rehabilitation, and better elastic properties (Wang et al., 2011; Song et al., 2014; Wang C. et al., 2018). Moreover, it has achieved satisfactory efficacy in early clinical application.

This study successfully simulated the surgical processes of MNSF treated using two different fixations (LSIF and TSEF). The maximum ROM and the maximum stresses on screws, rods, fractured vertebral bodies, and intervertebral discs of the two models were compared under six conditions (flexion, extension, left and right bending, and left and right rotation). In conclusion, we find that TSEF shows the better biomechanical characteristics in ROM preservation and discs stress distribution compared to LSIF. TSEF reduces the stress concentration of the connecting rods and the stress shielding of the fractured vertebral bodies. Therefore, the TSEF might be a better alternative for MNSF.

## Data Availability

The original contributions presented in the study are included in the article/Supplementary material, further inquiries can be directed to the corresponding author.
